# Argon does not affect cerebral circulation or metabolism in male humans

**DOI:** 10.1371/journal.pone.0171962

**Published:** 2017-02-16

**Authors:** Frank Grüne, Stephan Kazmaier, Sanne Elisabeth Hoeks, Robert Jan Stolker, Marc Coburn, Andreas Weyland

**Affiliations:** 1 Department of Anaesthesiology, Erasmus University Medical Centre, Rotterdam, The Netherlands; 2 Centre of Anaesthesiology, Critical Care, Emergency Medicine and Pain Therapy (ZARI), University-Hospital of Göttingen, Göttingen, Germany; 3 Department of Anaesthesiology, University Hospital RWTH Aachen, Aachen, Germany; 4 Department of Anaesthesiology, Critical Care, Emergency Medicine and Pain Therapy, Klinikum Oldenburg, Medical Campus University of Oldenburg, Oldenburg, Germany; Boston University, UNITED STATES

## Abstract

**Objective:**

Accumulating data have recently underlined argon´s neuroprotective potential. However, to the best of our knowledge, no data are available on the cerebrovascular effects of argon (Ar) in humans. We hypothesized that argon inhalation does not affect mean blood flow velocity of the middle cerebral artery (Vmca), cerebral flow index (FI), zero flow pressure (ZFP), effective cerebral perfusion pressure (CPPe), resistance area product (RAP) and the arterio-jugular venous content differences of oxygen (AJVDO_2_), glucose (AJVDG), and lactate (AJVDL) in anesthetized patients.

**Materials and methods:**

In a secondary analysis of an earlier controlled cross-over trial we compared parameters of the cerebral circulation under 15 minutes exposure to 70%Ar/30%O_2_ versus 70%N_2_/30%O_2_ in 29 male patients under fentanyl-midazolam anaesthesia before coronary surgery. Vmca was measured by transcranial Doppler sonography. ZFP and RAP were estimated by linear regression analysis of pressure-flow velocity relationships of the middle cerebral artery. CPPe was calculated as the difference between mean arterial pressure and ZFP. AJVDO_2_, AJVDG and AJVDL were calculated as the differences in contents between arterial and jugular-venous blood of oxygen, glucose, and lactate. Statistical analysis was done by t-tests and ANOVA.

**Results:**

Mechanical ventilation with 70% Ar did not cause any significant changes in mean arterial pressure, Vmca, FI, ZFP, CPPe, RAP, AJVDO_2_, AJVDG, and AJVDL.

**Discussion:**

Short-term inhalation of 70% Ar does not affect global cerebral circulation or metabolism in male humans under general anaesthesia.

## Introduction

Argon is the longest known rare gas of the group of noble gases. Compared to xenon, it has a higher natural abundance in the atmosphere (gas fraction of dry air at sea level: 20.9% oxygen, 78.1% nitrogen, 0.9% argon, 0.03% carbon dioxide, helium 0.000005%, and 0.00000009% xenon) [1]. It can be obtained as a pure gas of pharmaceutical quality at low costs. Stable argon gas is known to be inert. It does not change vital parameters and has no anaesthetic properties at sea level [2,3]. When argon is administered by inhalation it does not require complex ventilator settings. Inhalation of argon/oxygen mixtures is used in humans to measure coronary [4] and cerebral blood flow [5,6]. Argons beneficial neuroprotective and organoprotective properties have been observed in animal experiments *in vitro* and *in vivo*, but rarely in human studies [7,8].

Up to now the cerebrovascular and cerebrometabolic effects of argon have not been investigated in humans, which may be essential for a possible future clinical application of argon as an organoprotective agent. We performed a larger series of clinical studies using an argon inhalation method for measurements of global cerebral blood flow (CBF), a modification of the Kety-Schmidt technique. In a prospective, controlled, cross-over study design, we investigated the effects of hyperventilation versus hypoventilation in anesthetized patients on parameters of circulation and cerebral metabolism, which in part has been recently published [9]. In the same group of patients we also investigated the short-term effects of argon inhalation. We hypothesized that argon has no effects on parameters of cerebral blood flow velocity, cerebrovascular perfusion pressure, blood gas analysis, and global cerebral metabolism.

The original rationale for this substudy was a methodological question, as we wanted to rule out the possibility, that our method of CBF measurement (which required argon inhalation) *per se* might have any influence on the cerebrovascular and cerebrometabolic variables. Because of the recent interest in potential organoprotective properties of argon, we believe that these data should be made accessible to the scientific community.

## Materials and methods

### Design

In this prospective controlled study we investigated the cerebrovascular CO_2_-reactivity of CBF, cerebral blood flow velocity of the middle cerebral artery (Vmca), and cerebral metabolic rate of oxygen, glucose and lactate (CMRO_2_, CMRG, CMRL) in 30 patients before cardiovascular surgery at the University of Göttingen Medical Centre, Germany. CBF was measured using the modified Kety-Schmidt inert gas saturation technique with stable argon gas as an indicator (70% Ar / 30% O_2_ gas mixture). Vmca was simultaneously measured by transcranial Doppler sonography (TCD) [9]. Subsequently, after this investigation of hypoventilation versus hyperventilation on CBF, Vmca and CMR, we divided the 30 patients into 3 subgroups (10 patients per group) for further examinations in the same setting. With additional measurements in these patients we studied the cerebrovascular effects (i) of nitroglycerine [10], (ii) of halothane [11], and (iii) of more extensive variations of PaCO_2_ [12].

For quality control purposes, we also studied in these patients the cerebrovascular and metabolic effects of argon itself at the beginning of each series of CBF measurements. The primary rationale for this analysis was to investigate if our method of CBF measurement (argon saturation method) *per se* has any influence on cerebrovascular circulation or metabolism. Thus haemodynamic and laboratory data, which have been used to calculate variables of the first point of measurement in previous publications, are partly used in this analysis [9–12].

Cerebral flow index (FI), effective cerebral perfusion pressure (CPPe), cerebral zero flow pressure (ZFP), and the inverse slope of pressure-flow velocity relationships (resistance area product, RAP) were calculated in addition to standard variables from transcranial Doppler and haemodynamic recordings.

The study project followed the recommendations of the Declarations of Helsinki and the European Union Commission and European Medicines Agency (Council Directive 91/507/EEC and 75/318/EEC). Ethical approval for this study was provided by the Medical Ethical Committee of the Georg-August-University of Göttingen, Göttingen, Niedersachsen, Germany (Trial: Validation of transcranial Dopplersonography as a monitoring technique of the cerebral circulation during general anaesthesia (Validierung der transkraniellen Dopplersonographie als Überwachungsverfahren der zerebralen Hämodynamik unter anästhesiologischen Bedingungen), Ethical Committee N° 07/09/90). The trial has been retrospectively registered at the German Clinical Trials Registry (No.: DRKS00011535).

### Endpoints

The endpoints of the study were changes in the mean Vmca, FI, CPPe, ZFP, RAP, and the arterio-jugularvenous content differences of oxygen (AJVDO_2_), glucose (AJVDG), and lactate, (AJVDL). We hypothesized that in patients under intravenous anaesthesia short-term inhalation of 70% argon does not alter cerebral haemodynamics or metabolism.

### Inclusion

Due to logistical reasons and funding we could perform only 1–2 measurements per month. Thus, standard-screening procedures could not be applied in this cross-over trial. Patients were eligible for inclusion if scheduled for elective coronary surgery. Exclusion criteria were: age older than 80 years, active neurological disease, and a history of cerebrovascular disease, brain injury, or intracranial surgery.

Transcranial Doppler measurements of Vmca from the transtemporal window fail with above average incidence in elderly female patients due to less mineral bone density and subsequently reduced ultrasound penetration [13]. Therefore, we included only male patients in this study. All patients were informed about the purpose of the study and provided written informed consent before being enrolled. None of the eligible patients refused inclusion in the trial.

### Anaesthesia procedure

Individual medications were continued until surgery. Intravenous anaesthesia was induced and maintained by continuous intravenous administration of fentanyl, midazolam and pancuronium. All patients received two peripheral intravenous lines, an endotracheal tube, a nasogastric tube, and a urine catheter. After arterial, central venous and pulmonary artery catheterization a jugular bulb catheter was inserted by retrograde puncture of the right internal jugular vein. The correct position of the jugular bulb catheter tip was verified by fluoroscopy to prevent inadvertent extracerebral contamination of blood samples. The withdrawal rate of blood samples used for blood gas analysis, metabolic tests and for gas chromatographic determination of the argon concentration at the end of the saturation period was 5 mL / 20 s [14]. The anaesthesia procedure, the details of mechanical ventilation and the methods of catheter insertion have been described in previous reports in detail [5,9].

### Measurements and calculations

Measurements were performed in 30 patients after induction of general anaesthesia and before surgery during haemodynamic and respiratory steady-state conditions.

First measurements were performed under 70%N_2_/30%O_2_, then ventilation was switched to 70% argon/30%O_2_ mixture for 15 minutes. The ventilator settings have been adjusted following to our study protocol. All patients have been ventilated by two identical anaesthesia machines. When starting the argon-wash-in-period we changed the ventilators, which had been prefilled with the respective gas mixture, using identical respiratory settings and identical inspired oxygen fraction. End-expiratory CO_2_ concentrations were continuously recorded to ensure a stable PaCO_2_ during argon ventilation.

Blood samples were drawn twice, at the beginning (baseline) and end of the argon wash-in period (argon), to measure variables of blood gas analysis (ABL; Radiometer, Copenhagen, Denmark), glucose and lactate.

Blood flow velocity in the proximal (M1) segment of the middle cerebral artery (Vmca) was measured by TCD. To ensure a constant position of the ultrasound probe during the investigation period, we used a probe holder (IMP2 monitoring probe holder, EME, Überlingen, Germany). During measurements the patients’ heads were fixed in midline position. Arterial blood pressure was measured invasively in the radial artery, ipsilateral to the TCD probe.

The analyses of the ZFP, CPPe and RAP have been performed after the study period. Cerebral ZFP was calculated from data at baseline and at the end of each argon gas saturation phase from two simultaneous 10 s recordings (two breathing cycles) of the Vmca curves and arterial pressure curves. Over each 10 s period we first averaged consecutive pairs of diastolic, mean and systolic data of ABP and Vmca. These data were used to generate a pressure/flow velocity plot. ZFP was then extrapolated by linear regression analysis of the ABP-Vmca relationship. The ABP axis intercept of the regression line determines the ZFP [15,16]. The cerebral ZFP was used as a measure of the effective downstream pressure of the cerebral circulation. Consequently, CPPe was calculated as CPPe = mean ABP-ZFP. In the relationship between ABP and Vmca, the RAP is defined as the inverse slope of their linear regression line [17,18].

Inside the spectral envelope each velocity has an intensity (usually color-coded on the display), which is proportional to the volume of blood at this velocity. The flow related to that volume of blood is therefore proportional to the product of its velocity and acoustic intensity. Summing these different flows gives a flow index (FI), which is proportional to total flow. The FI was thus computed as the sum of each acoustic intensity within the TCD spectrum multiplied by the corresponding velocity. We averaged all signals over the entire waveform, which yields the mean FI for that particular 10 s interval [19–21].

The arterial-to-jugular venous concentration difference of a substance *x*, was calculated as AJVD(x) = Ca(x)—Cjv(x). We calculated the difference in arterio-jugular venous content of oxygen (AJVDO_2_), glucose (AJVDG), and lactate (AJVDL) [22]. By definition, positive AJVD values indicate consumption or net influx, and negative values indicate production or net efflux. For AJVDL, in case of cerebral lactate production, we thus expected negative values.

### Statistical analysis

In this secondary analysis study setting a prior sample size calculation was not performed. Each patient served as his own control. The results presented are expressed as mean (standard deviation) unless otherwise stated. Normal distribution of data was assessed both visually with inspection of histograms and with the D'Agostino-Pearson omnibus K2 method. The differences between baseline and argon gas inhalation were calculated using t-tests for paired data or Welch-test and non-parametric Wilcoxon signed-rank test, if indicated [23]. To provide an estimate of the effect of argon gas and its clinical meaningfulness, we calculated mean differences (MD) and their 95 per cent confidence intervals (MD; 95% CI upper bound, lower bound; *P*-value) [24]. In case of significant differences between the baseline and the argon period, all primary endpoints were additionally tested by one-way ANOVA for repeated measurements followed by Bonferoni multiple comparison tests in order to prevent type I error [25].

All statistical analyses were performed two-sided and a p-value of p< 0.05 was considered to be significant. Database sheets were done by MS Excel^®^ for Mac 2011 (Microsoft, Redmond, Washington, USA). Statistical procedures and graphs were made using Prism 6.0 (GraphPad Software, La Jolla, California, USA).

## Results

The study period was 27 months (February 20, 1991 until May 10, 1993). A total of 30 male patients were examined. In one patient an unexpected sudden increase in heart rate and blood pressure occurred after an unintended surgical stretcher movement during the recordings. This was interpreted as a short phase of insufficient depth of anaesthesia, which ceased following an additional bolus of fentanyl and midazolam. The patient was thereafter completely excluded from further analysis because we assumed insufficient steady state conditions.

The mean TCD-insonation depth of the MCA was 51 (3) mm. TCD signals were of high quality in all patients except for two patients during the baseline measurement (patient 1 and 5). These data have been excluded from respective analysis.

Mean age of the included 29 patients was 56 (6) yrs. (median 57, range 41–65 yrs.), mean height 174 (6) cm, and mean body weight 78 (8) kg. One of our patients had diabetes mellitus type 2, treated with biguanides. None of the patients showed increased levels of blood glucose. A minimal, but significant decrease of mean blood temperature between the two periods of measurements was observed (Temp baseline 35.6 (0.5)°C, argon 35.5 (0.5)°C, MD 0.1, 95% CI 0.07 to 0.2, P< 0.01). There was a minimal decrease in heart rate (HR) baseline 58 (8) bpm and argon 56 (8) bpm, MD 2; 95% CI 1 to 2, P < 0.01), arterial oxygen saturation (SaO_2_ baseline 97 (1)% and argon 96 (2)%, MD 1, 95% CI 1 to 1, P < 0.01), and arterial partial pressure of oxygen (PaO_2_ baseline 139 (42) mmHg and argon 109 (26) mmHg, MD 29, 95% CI 17 to 41, P < 0.01). Arterial lactate concentration showed a very small but significant increase (Lac_ART_ baseline 0.59 (0.16) mmol·L^-1^ and argon 0.63 (0.17) mmol·L^-1^, MD -0.04, 95% CI −0.08 to -0.01, P 0.04). Levels of haemoglobin were unchanged before and after argon exposure (Hb baseline 12.7 (1.4) mg·dL^-1^ and argon 12.7 (1.4) mg·dL^-1^, MD 0.01, 95% CI −0.06 to 0.08, P 0.84) as well as levels of jugular venous oxygen saturation (SjvO_2_ baseline 50 (10)% and argon 50 (11)%, MD 0.44, 95% CI −0.78 to 1.67, P 0.46).

When comparing inhalation of 70% N_2_ / 30% O_2_ with 70% Ar / 30% O_2_ there were no significant and clinically relevant changes in mean arterial pressure (MAP), mean Vmca, FI, ZFP, CPPe, RAP AJVDO_2_, AJVDG and AJVDL.

**Fig 1:**
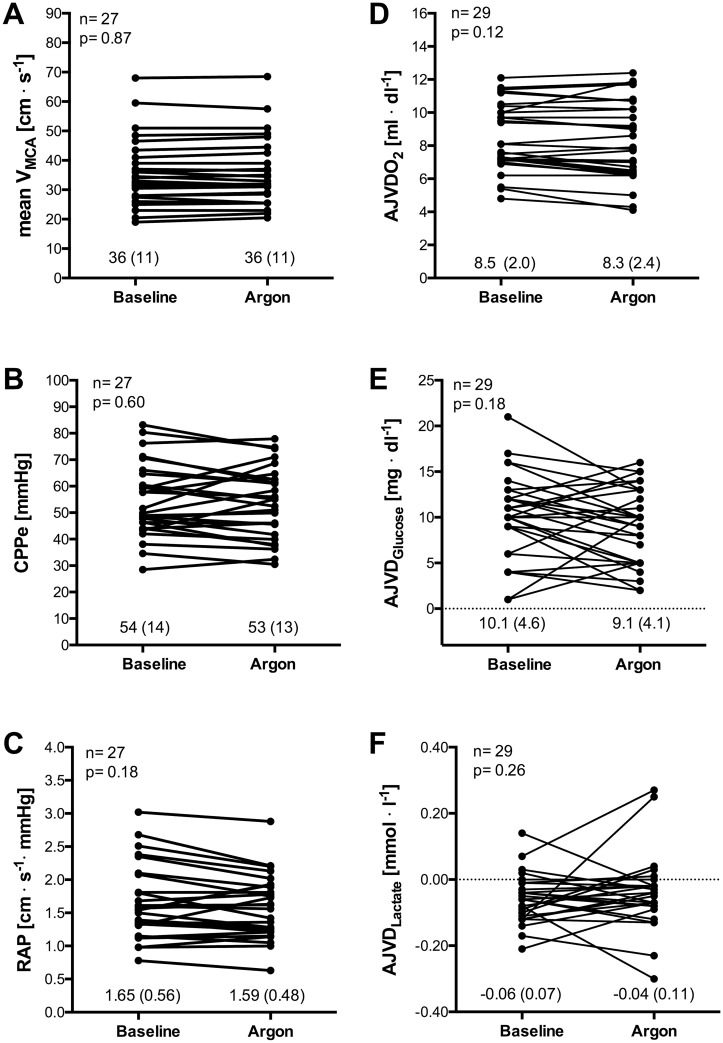
Effect of argon inhalation on cerebral circulation and metabolism. Values of mean blood flow velocity of the middle cerebral artery (Vmca), effective cerebral perfusion pressure (CPPe), resistance area product (RAP), arterio-jugular venous differences in oxygen (AJVDO_2_) glucose (AJVDG), and lactate (AJVDL) during baseline (70%N_2_/30%O_2_) compared with argon inhalation (70%Ar/30%O_2_). *Straight lines* link individual values for each subject. Data are mean (SD) for each measurement. The P values, which refer to the difference between two measurement points, were calculated using two-sided t-test for paired data. Due to artefacts during TCD recording, data of patient 1 and 5 have been excluded from respective analysis § n = 28, * n = 27.

The most important haemodynamic and metabolic data are presented in Table 1 and Fig 1. There were no relevant changes in other basic haemodynamic and blood gas variables between the baseline and argon inhalation period (see S1 Table and S1 File in the supplemental data content).

**Table 1:** Haemodynamic and metabolic data.

	Baseline		Argon					n = 29
Variable	mean	SD	mean	SD	Dimension	MD	CI limits	P
meanV_MCA_	36	11	36	11	[cm·s^-1^]	0	(-0.5; 0.4)	0.87*
FI ^#$^	154	84	147	74	[]	7	(-4; 17)	0.20*
MAP	74	12	73	13	[mmHg]	1	(-1; 3)	0.33^§^
ZFP ^#$^	20	8	20	8	[mmHg]	0	(-2; 2)	0.91*
CPPe	54	14	53	13	[mmHg]	1	(-2; 4)	0.60*
RAP ^#$^	1.65	0.56	1.59	0.48	[mmHg·s·cm^-1^]	0.06	(-0.03; 0.15)	0.18*
AJVDO_2_	8.5	2.0	8.3	2.4	[ml dL^-1^]	0.2	(-0.1; 0.4)	0.12
AJVDG^#^	10.1	4.6	9.1	4.1	[mg dL^-1^]	1.1	(-0.5; 2.6)	0.18
AJVDL^#$^	-0.06	0.07	-0.04	0.11	[mmol L^-1^]	0.02	(-0.07; 0.02)	0.26

Baseline = inhalation of 70% N_2_ / 30% O_2_. Argon = inhalation with 70% Ar / 30% O_2_. The P values, which refer to the difference between two measurement points, were calculated using two-sided t-test for paired data. Because the variances of some outcome variables differed between two measurement points, these parameters were additionally examined by Welch test (#) and nonparametric Wilcoxon signed-rank test ($) (P<0.05). Due to artefacts during TCD or blood pressure recording, data of patient 1 and 5 have been excluded from respective analysis § n = 28, * n = 27.

AJVDG = arterio-jugular venous difference in glucose; AJVDL = arterio-jugular venous difference in lactate; AJVDO_2_ = arterio-jugular venous difference in oxygen, CI = confidence interval (5%; 95%), CPPe = effective cerebral perfusion pressure defined as MAP-ZFP, FI = Flow index, MAP = mean arterial pressure, MD = mean differences, RAP = resistance area product, SD = standard deviation, mean Vmca = mean blood flow velocity of the middle cerebral artery, ZFP = zero flow pressure.

Ventilation parameters and PaCO_2_ levels of the patients were effectively kept constant (mean PaCO_2_ baseline = 36 (5) mmHg, (median 34, range 28 to 45), mean PaCO_2_ argon = 37 (7) mmHg (median 34, range 26 to 50). The mean difference of PaCO_2_ between the baseline and the argon measurements was minimal MD = −0.6 (2.4), CI −1.5 to 0.36 mmHg, P 0.22 (median differences 0.0, P 0.46).

## Discussion

We investigated the effects of an argon-oxygen gas mixture on cerebral circulation and metabolism in cardiovascular patients under fentanyl-midazolam anaesthesia. The most prominent result of our study is that no significant and clinically relevant changes were found in Vmca, FI, CPPe, ZFP, RAP, AJVDO_2_, AJVDG and AJVDL when comparing inhalation of 70% Ar / 30% O_2_ with 70% N_2_ / 30% O_2_

To the best of our knowledge, this study is the first report about cerebrovascular and cerebrometabolic effect of argon in humans. Most of the data on biological effects of argon has been accomplished by *in vitro* and *in vivo* animal studies, but rarely in humans. Medical research in the 1930s among military divers was one of the earliest observations of the biological effects of argon. Argon is well known to be inert at standard gas conditions. Mental impairment at high pressures had been observed [26, 27]. One trial in humans examined the long-term effects (up to 9 days) under hyperbaric argon atmosphere, which showed no negative influence on work performance [28]. These data suggested a shift in lipid metabolism and an increased resistance to hypoxic hypoxia under argon atmosphere. Other studies observed an increase of oxygen consumption under argon, which was interpreted as a catalytic activity of argon on oxygen kinetics [29]. Recently, Alderliesen et al. explored the potential effect of argon ventilation on important physiological parameters in newborn piglets. Argon exposure up to 80% did not change heart rate, arterial blood pressure, rectal temperature, arterial oxygen saturation, amplitude-integrated electro-encephalogram and regional cerebral oxygen saturation measured by near infrared spectrometry [3]. Our data in humans support these former observations that argon inhalation did not cause relevant changes in basic vital parameters.

Transcranial Doppler sonography measures flow velocity in the basal cerebral arteries. Today, Vmca and its indices are routinely used to assess components of cerebral circulation. Although Vmca is not a direct measure of CBF, changes in flow velocity generally correlate well with changes in CBF, except for specific situations, which may affect MCA diameter such as vasospasm, hypercapnia, migraine attacks, nitroglycerine, or other vasoactive agents [30–32]. In our study we additionally monitored the sonographic flow index, which showed consistent results when compared with Vmca. We thus have no reason to assume that a possible change in CBF during argon exposure has not been detected by our TCD measurements because of methodological limitations.

The noble gas xenon has excellent narcotic properties and may affect cerebral circulation [33, 34]. Trials about the effect of xenon on CBF showed inconsistent results in animals and humans [35–39]. The dose of xenon inhalation, however, seems to play an important role. In monkeys, subanaesthetic xenon (35%) caused a global reduction in CBF [37], whereas anaesthetic concentration (80%) increased CBF by 53% [40]. Using the 133-xenon clearance method, subanaesthetic (35%) xenon was shown to increase human rCBF by approximately 12% [41]. A recent investigation under steady conditions showed that anaesthetic concentration of xenon (65%) decreased regional CBF in several brain regions in animals and in humans. The greatest decreases were detected in the cerebellum (-35%), the thalamus (-23%) and the parietal cortex (-11%) [42]. However, xenon anaesthesia increases flow velocity of basal cerebral arteries by about 30% [43,44]. The inconsistency of these results may partly be explained by methodological reasons such as inconstant carbon dioxide levels, different anaesthetic agents and by interspecies differences in anaesthetic concentrations for xenon in animals and in humans [42]. Argon has, in contrast to xenon, no anaesthetic properties at sea level [45]. Up to now there are no reports about the effects of argon on cerebral circulation obtained by TCD. Our data demonstrate that inhalation of 70% argon in humans did not affect Vmca or cerebral FI.

Cerebral ZFP, CPPe and RAP are supplemental determinants of cerebral blood flow. Using the CPPe as the driving pressure of the cerebral circulation does not require invasive measurements of intracranial pressure (ICP) and offers advantages in understanding pathophysiology, because changes of the effective downstream pressure irrespective of ICP will also be reflected by this kind of calculation [12,15]. The inverse of the slope of the arterial blood pressure-Vmca plot, the RAP, has been used as index of cerebrovascular resistance [12]. These less invasive methods of assessing cerebral perfusion are well established and have improving accuracy [46]. In our study, inhalation of argon showed no clinically relevant changes in ZFP, CPPe, and RAP.

Reports on the effect of argon on CBF and parameters of cerebral metabolism are not available up to now. Global cerebral hypoperfusion often is defined as a SjvO_2_ less than 50% whereas cerebral ischemia is assumed when SjvO_2_ is less than 40% and AJVDO_2_ is greater than 9 ml·dL^-1^ [47]. Total intravenous anaesthesia with fentanyl/midazolam in humans causes a moderate proportional reduction in CBF due to a decrease in both cerebrovascular resistance and CMRO_2_ [5, 48]. Thus, a constant SjvO_2_ and unchanged arterio-jugular venous content differences of oxygen, glucose, and lactate (AJVDO_2_, AJVDG, AJVDL) suggest intact flow-metabolic coupling of the brain [22, 49]. In contrast to intravenous anaesthesia, volatile anaesthetics cause a partial uncoupling of CBF and metabolism because of a direct cerebral vasodilatatory effect [50, 51]. In our patients inhalation of 70% argon showed no clinically relevant changes in SjvO_2_ and/or arterio-jugular venous content difference of oxygen, glucose, and lactate. The coupling of cerebral flow and metabolism thus seems to be unchanged during argon exposure and our findings indicate a constant cerebral metabolic rate of oxygen and glucose.

Despite the increasing comprehension of neuroplasticity and neuroprotection, the exact mechanisms by which argon improves outcome remain far from being understood [52]. Besides an interference with neuronal ion-gated channels and cellular signalling pathways as well as anti-apoptotic effects, the modulation of neuroinflammation seems to play a crucial role [53]. Neuroprotective effects of argon were observed at different concentrations (25%, 50% and 74%) in both *in vitro* models of cerebral ischemia and traumatic brain injury [54]. Brücken and colleagues demonstrated in rats that one hour of 70% argon inhalation after cardiac arrest provided a significant reduction in histopathological damage of the neocortex and hippocampus, and was associated with a marked improvement in functional neurological recovery. At baseline, as well as after 4 hours after cardiac arrest, no significant differences were observed with regard to haemodynamics, variables of gas exchange, or lactate and glucose concentrations between groups [55]. In a postresuscitation treatment study in pigs, 70% argon inhalation lead to a fast and complete neurological recovery 72 hours after cardiopulmonary resuscitation. In perinatal asphyxia animal models with piglets, ventilation with up to 80% argon during normoxia, and 50% argon after hypoxia did not affect heart rate, blood pressure, cerebral saturation and electrocortical brain activity [3].

Our study was not designed to assess the neuroprotective effect of argon ventilation. Based on our observations and the previous, safe use of argon in adults in the past, as well as the efficacy studies described above, further safety studies in humans (e.g. after cardiac arrest, cerebral ischemia, traumatic brain injury, or even in neonates with perinatal asphyxia) appear warranted.

Some methodological aspects of our study have to be considered.

The type of anaesthesia may have potential influence on the results of our study. We have no reason to assume that intravenous anaesthesia with fentanyl and midazolam *per se* may have confounded our results. However, the results of this study cannot *a priori* be extrapolated to other types of anaesthesia. Similarly, the external validity of our data could be limited by the fact that some of our patients with coronary artery disease might be suffering from concomitant asymptomatic cerebrovascular disease.

Unexpectedly, there was a very slight reduction in SaO_2_ and PaO_2_. All patients have been ventilated by two identical anaesthesia machines. When starting the argon-wash-in-period we changed the ventilators, using identical respiratory settings and identical inspired oxygen fraction. The mild decrease in SaO_2_ and PaO_2_ might be related to the short discontinuation of PEEP during this manoeuvre. Fortunately, our measurements did not affect general conditions in our patients. All surgical procedures were without complications. All patients had been discharged from ICU within 2 days. There were no suspected unexpected serious adverse reactions (SUSARS) or serious adverse events (SAE).

### Conclusion

In male cardiac surgical patients under fentanyl-midazolam anaesthesia, short-term ventilation with argon (70%Ar / 30% O_2_) did not show any effects on the cerebral circulation or on global oxygen and glucose metabolism. The lack of cerebrovascular and cerebrometabolic effects suggests future studies on the use of argon which should confirm the safety of argon inhalation during longer periods and may investigate the organ protective effects of argon in humans.

## Supporting information

S1 File Study data file. No legend.
(XLSX)

S1 Table Effect of argon on parameters of blood gas analysis and haemodynamics. Baseline = inhalation of 70% N_2_ / 30% O_2_. Argon = inhalation with 70% Ar / 30% O_2_. The P values, which refer to the difference between two measurement points, were calculated using two-sided t-test for paired data (* P<0.05). Due to artefacts during blood pressure recording, data of patient 1 has been excluded from respective analysis § n = 28. CI = confidence interval (5%; 95%), Gluc_ART_ = arterial blood glucose concentration, Gluc_JV_ = jugular bulb blood glucose concentration, Hb = haemoglobin concentration, Hct = haematocrit, HR = heart rate in beats per minute, K = serum potassium concentration, Lac_ART_ = arterial blood lactate concentration, Lac_JV_ = jugular bulb blood lactate concentration, MD = mean differences, MAP = mean arterial blood pressure, Na = serum sodium concentration, pH_ART_ = pH of arterial blood, pH_JV_ = pH of jugular bulb blood, PaCO_2_ = arterial partial pressure of CO_2_, PjvCO_2_ = jugular bulb partial pressure of CO_2_, PaO_2_ = arterial partial pressure of O_2_, PjvO_2_ = jugular bulb partial pressure of O_2_, SaO_2_ = arterial blood oxygen saturation, SjvO_2_ = jugular bulb blood oxygen saturation, SBC = standard bicarbonate concentration, SD = standard deviation.
(DOCX)
