# Supplementary material for: Argon does not affect cerebral circulation or metabolism in male humans
Source: PLoS One. 2017 Feb 16;12(2):e0171962. doi: 10.1371/journal.pone.0171962 (PMC5313187; doi:10.1371/journal.pone.0171962)
Supplement: S1 Table — Baseline = inhalation of 70% N2 / 30% O2. Argon = inhalation with 70% Ar / 30% O2. The P values, which refer to the difference between two measurement points, were calculated using two-sided t-test for paired data (* P<0.05). Due to artefacts during blood pressure recording, data of patient 1 has been excluded from respective analysis § n = 28. CI = confidence interval (5%; 95%), GlucART = arterial blood glucose concentration, GlucJV = jugular bulb blood glucose concentration, Hb = haemoglobin concentration, Hct = haematocrit, HR = heart rate in beats per minute, K = serum potassium concentration, LacART = arterial blood lactate concentration, LacJV = jugular bulb blood lactate concentration, MD = mean differences, MAP = mean arterial blood pressure, Na = serum sodium concentration, pHART = pH of arterial blood, pHJV = pH of jugular bulb blood, PaCO2 = arterial partial pressure of CO2, PjvCO2 = jugular bulb partial pressure of CO2, PaO2 = arterial partial pressure of O2, PjvO2 = jugular bulb partial pressure of O2, SaO2 = arterial blood oxygen saturation, SjvO2 = jugular bulb blood oxygen saturation, SBC = standard bicarbonate concentration, SD = standard deviation. (DOCX) [file pone.0171962.s002.docx]

# Supplemental Digital Content

## S1 Table. Effect of argon on parameters of blood gas analysis and haemodynamics.

|  | **Baseline** |  |  | **Argon** |  |  |  |  |  |
| --- | --- | --- | --- | --- | --- | --- | --- | --- | --- |
| **Variable** | **mean** | **SD** |  | **mean** | **SD** | **Dimension** | **MD** | **CI limits** | **P** |
|  |  |  |  |  |  |  |  |  |  |
| HR* | 58 | 9 |  | 56 | 9 | [bpm] | 2 | (1; 2) | <0.01^§^ |
| MAP | 74 | 12 |  | 73 | 13 | [mmHg] | 1 | (-1; 3) | 0.33^§^ |
| Hb | 12.7 | 1.4 |  | 12.7 | 1.4 | [ml dL^-1^] | 0.01 | (-0.1; 0.1) | 0.84 |
| Hct | 38 | 4 |  | 38 | 4 | [%] | 0.1 | (-0.1; 0.4) | 0.33 |
| Na | 143 | 2 |  | 143 | 2 | [mmol L^-1^] | 0 | (0; 0) | 0.57 |
| K | 4.3 | 0.4 |  | 4.3 | 0.3 | [mmol L^-1^] | -0.01 | (-0.13; 0.11) | 0.82 |
| Gluc _ART_ | 77 | 9 |  | 77 | 9 | [ml dL^-1^] | 1 | (-1; 2) | 0.56 |
| Gluc _JV_ | 67 | 8 |  | 67 | 8 | [ml dL^-1^] | -1 | (-2; 1) | 0.51  ** |
| Lac _ART_* | 0.59 | 0.16 |  | 0.63 | 0.18 | [mmol L^-1^] | -0.04 | (-0.08; -0.01) | 0.04 |
| Lac _JV_ | 0.65 | 0.15 |  | 0.67 | 0.20 | [mmol L^-1^] | -0.02 | (-0.04; 0.01) | 0.21 |
| PaO_2_* | 139 | 42 |  | 109 | 29 | [mmHg] | 29 | (17; 41) | <0.01 |
| PjvO_2_ | 30 | 6 |  | 30 | 7 | [mmHg] | 0 | (-1; 1) | 0.45 |
| SaO_2_* | 97 | 1 |  | 96 | 2 | [%] | 1 | (1; 1) | <0.01 |
| SjvO_2_ | 50 | 10 |  | 49 | 11 | [%] | 0 | (-1; 2) | 0.48 |
| PaCO_2_ | 36 | 5 |  | 37 | 7 | [mmHg] | -1 | (-1; 0) | 0.24 |
| PjvCO_2_ | 47 | 5 |  | 47 | 6 | [mmHg] | 0 | (-1; 1) | 0.68 |
| pH _ART_ | 7.43 | 0.06 |  | 7.43 | 0.07 | [ ] | 0.01 | (-0.01; 0.01) | 0.94 |
| pH _JV_ | 7.36 | 0.04 |  | 7.36 | 0.05 | [ ] | 0.01 | (-0.01; 0.01) | 0.43 |
| SBC _ART_ | 25.4 | 1.1 |  | 25.5 | 1.4 | [mmol L^-1^] | -0.1 | (-0.5; 0.2) | 0.50 |
| SBC _JV_ | 25.1 | 1.2 |  | 25.0 | 1.0 | [mmol L^-1^] | 0.1 | (-0.2; 0.4) | 0.60 |
|  |  |  |  |  |  |  |  |  |  |
